# Overexpression of cholinergic receptor nicotinic gamma subunit inhibits proliferation and differentiation of bovine preadipocytes

**DOI:** 10.5713/ab.22.0144

**Published:** 2022-09-07

**Authors:** Jiawei Du, Hui Zhao, Guibing Song, Yuan Pang, Lei Jiang, Linsen Zan, Hongbao Wang

**Affiliations:** 1 College of Animal Science and Technology, Northwest A&F University, Yangling, 712100, China; 2 National Beef Cattle Improvement Center, Northwest A&F University, Yangling, 712100, China

**Keywords:** *CHRNG*, Differentiation, Preadipocytes, Proliferation

## Abstract

**Objective:**

Muscle acetylcholine receptors have five alpha subunits (α, β, δ, ɛ, or γ), and cholinergic receptor nicotinic gamma subunit (CHRNG) is the γ subunit. It may also play an essential role in biological processes, including cell differentiation, growth, and survival, while the role of CHRNG has not been studied in the literature. Therefore, the purpose of this study is to clarify the effect of CHRNG on the proliferation and differentiation of bovine preadipocytes.

**Methods:**

We constructed a CHRNG overexpression adenovirus vector and successfully overexpressed it on bovine preadipocytes. The effects of CHRNG on bovine preadipocyte proliferation were detected by Edu assay, cell counting Kit-8 (CCK-8), real-time fluorescence quantitative polymerase chain reaction (RT-qPCR), Western blot and other techniques. We also performed oil red O, RT-qPCR, Western blot to explore its effect on the differentiation of preadipocytes.

**Results:**

The results of Edu proliferation experiments showed that the number of EDU-positive cells in the overexpression group was significantly less. CCK-8 experiments found that the optical density values of the cells in the overexpression group were lower than those of the control group, the mRNA levels of proliferating cell nuclear antigen (PCNA), cyclin A2 (CCNA2), cyclin B1 (CCNB1), cyclin D2 (CCND2) decreased significantly after CHRNG gene overexpression, the mRNA levels of cyclin dependent kinase inhibitor 1A (CDKN1A) increased significantly, and the protein levels of PCNA, CCNB1, CCND2 decreased significantly. Overexpression of CHRNG inhibited the differentiation of bovine preadipocytes. The results of oil red O and triglyceride determination showed that the size and speed of lipid droplets accumulation in the overexpression group were significantly lower. The mRNA and protein levels of peroxisome proliferator activated receptor gamma (PPARγ), CCAAT enhancer binding protein alpha (CEBPα), fatty acid binding protein 4 (FABP4), fatty acid synthase (FASN) decreased significantly.

**Conclusion:**

Overexpression of CHRNG in bovine preadipocytes inhibits the proliferation and differentiation of bovine preadipocytes.

## INTRODUCTION

Peoples’ quality of life is improving year by year, so the demand for beef is also increasing [1]. Beef is one of the main meats eaten by humans. It is favoured by consumers because of its rich protein content, unique taste, and flavour. The composition of fat and fatty acids in fat or muscle tissue has a positive effect on the appearance, texture, flavour, hardness, and shelf life of meat. It is an important part that affects the taste and flavor [2]. At present, the study of adipose tissue of beef cattle, especially the analysis of the mechanism of proliferation, differentiation, and apoptosis of adipocytes, is the key to improve the breed and industrial development of beef cattle in China.

Adipose tissue is almost all over the animal body and has strong plasticity in the whole life process; it is the central energy storage of the body and plays an essential role in energy metabolism and balance [3,4]. Adipocytes are the basic unit of adipose tissue, and their proliferation and differentiation ability are closely related to fat deposition ability. Intramuscualr fat (IMF) is one of the key factors affecting beef quality, its content affects the juiciness, flavor and tenderness of beef [5]. The number of adipocytes in the body is mainly determined by the proliferation of adipocytes [6]. The differentiation of preadipocytes is closely related to the cell cycle. Contact inhibition occurs after cell convergence, withdraws from the cell cycle and turns to cell differentiation [7]. The deposition of fat includes the processes of proliferation, differentiation, and apoptosis of adipocytes, which are regulated by a variety of transcription factors and adipose secretion factors. Therefore, further understanding of the differentiation of preadipocytes may provide valuable information for improving the meat quality of livestock and poultry [8].

The cholinergic receptor nicotinic gamma subunit ( *CHRNG*) gene is a differentially expressed gene screened by transcriptome sequencing data in adipocytes in a preadipocyte-muscle cell co-culture system. Its expression is extremely low in adipocytes and significantly increased in adipocytes under co-culture (transcriptome sequencing data not yet published). Muscle nicotinic acetylcholine receptor (AChR) is a transmembrane protein with five different subunits, and CHRNG is one of the subunits [9]. According to reports that CHRNG can cause autosomal recessive Escobar syndrome [10,11]. CHRNG is vital for neuromuscular development and ligand binding [12]. The destruction of subunits *in vitro* prevents the correct localization of the receptor in the cell membrane [10]. Nicotinic AChR plays an important role in the regulation of systemic blood pressure [13]. It may also play an essential role in biological processes, including cell differentiation, growth and survival [14]. Gamma subunits promote neuromuscular signal transduction and are also important for neuromuscular histogenesis [15]. So far, the regulatory effect of CHRNG on adipocytes has not been reported. In this study, Qinchuan bovine preadipocytes were selected as research materials to explore the effect of CHRNG on the proliferation and differentiation of bovine preadipocytes at the cellular and molecular levels. With the aim to provide reference materials for further research on the role of this gene in the growth and development of Qinchuan cattle adipose tissue and the selection and improvement of Qinchuan cattle.

## MATERIALS AND METHODS

The bovine primary preadipocytes used in this experiment were all isolated from healthy newborn Qinchuan Cattle. The care and feeding of the animals used in this study were approved by the Institutional Animal Care and Use Committee of China (College of Animal Science and Technology, Northwest A&F University, China; No. 2013–23,20 April 2013). The implementation of the animal experimental procedures was performed in strict accordance with the guidelines of the Administration of Affairs Concerning Experimental Animals (Ministry of Science and Technology, China, 2004).

### Isolation, culture, and differentiation of bovine preadipocytes

Under sterile conditions, subcutaneous adipose tissue was taken and put into 1×phosphate buffer saline (PBS) containing 10% penicillin/streptomycin in newborn Qinchuan Cattle (3 days old). It was then removed from the aseptic console, separated from connective tissue and blood vessels with sterile tweezers and scissors, and digested with 0.25% collagenase I and 0.1% dispaseII for 1 to 2 hours. After digestion, filtered, and centrifuged for ten minutes, then the supernatant discarded. The cells were then pipetted and grown in complete medium (contains 15% fetal bovine serum [FBS; Gibco, New York, USA] and 1% penicillin/streptomycin), after 12 hours, cells were washed 3 times with 1×PBS to remove foreign cells, replaced with fresh DMEM/F12 (dulbecco’s modified eagle medium) complete medium and continued to culture, and the medium was changed every 1 d. To induce bovine preadipocyte differentiation, we used a growth medium containing 10% FBS, 0.5% insulin, 0.3%IBMX, 0.01% dexamethasone and 0.01% rosiglitazone. Two days later, we used a growth medium containing 10% FBS and 0.5% insulin to maintain bovine adipocyte differentiation.

### Adenovirus infects bovine preadipocytes

The viral vector used in this experiment was obtained by amplifying the full-length sequence of CHRNG CDS (NM_174273.2) and inserting it into the adenovirus vector pHBAd-CMV-EGFP. The objective shuttle plasmid *CHRNG* and adenovirus cytoskeleton plasmid were cotransfected into HEK293 cells to obtain *CHRNG* virus, which was amplified and purified, and then the titer was determined, the control was an empty vector virus without the CDS region of the target gene (This step was completed by Shanghai Heyuan Biological Company). Determined the multiplicity of infection, in adipocytes and infected the adipocytes when they had grown to 80%, and changed the standard growth medium 12 hours later.

### Oil red O staining and determination of triglycerides

The cells were washed with PBS, then fixed with formaldehyde for 30 minutes, then washed with PBS for three times, stained with oil red O working solution (0.5 g oil red O [Sigma-Aldrich, Shanghai, China], isopropanol 60 mL, double steamed water 40 mL) for 30 minutes, washed with PBS for three times, and finally photographed. When determining the content of triglyceride (Applygen company, Beijing, China), the content of triglyceride was collected by trypsin digestion cells, then the lysate was heated at 70°C for 10 minutes, the supernatant was obtained by centrifugation, and the working solution (R1:R2 = 4:1) was added to the supernatant. The glycerol standard sample was diluted in gradient and the standard curve was made. The optical density value of the sample was determined by enzyme labelling instrument (Infinite M200 pro), and the concentration of triglyceride was calculated.

### EDU proliferation assay

We used Cell-Light EDU Apollo567 in Vitro kit (RiboBio, GuangZhou, China) to detect the proliferation of bovine preadipocytes. According to the manufacturer’s agreement, the cells were incubated in EDU medium for 2 hours, and PBS washed the cells 1 to 2 times for 5 minutes each time. The cells were incubated in a fixed solution (PBS containing 4% paraformaldehyde) for 30 min. A 50 μL glycine solution was added to each well after discarding the fixed solution, washed with PBS for 5 minutes, and then incubated with 100 μL osmosis for 10 minutes. We added 100 μL staining reaction solution to each hole, and shaken for 30 minutes, then cleaned with PBS for three times, and 100 μL 1× Hoechst 33342 was added to hole to avoid light, room temperature, shaker incubation for 30 minutes, and PBS cleaning for three times.

### Cell counting kit 8 assay

The cells were inoculated into a 96-well plate for standard culture, 10% CCK8 solution (FC101; TRANS, Shang Hai, China) was added to each well, and the cells were cultured in the 37°C for 4 hours. The absorbance of 450 nm was measured by an enzyme labelling instrument.

### Real-time fluorescence quantitative polymerase chain reaction

Total cellular RNA was extracted with RNAiso Plus (Takara, Mountain View, CA, USA) reagent according to the instructions. Glyceraldehyde-3-phosphate dehydrogenase (GAPDH) was used as an internal reference for the polymerase chain reaction (PCR) reaction. The PCR reaction system 15 μL: TB Green II 7.5 μL, cDNA 1.2 μL, upstream and downstream primers 0.3 μL respectively, plus RNase free H2O complement system, the reaction was performed on Bio-Rad fluorescence quantitative PCR (CFX Connect). 2^−ΔΔCt^ method was used to analyze the data [16]. All the real-time fluorescence quantitative PCR (RT-qPCR) results in this study have *GAPDH* as the internal reference factor, and the sequence of all real-time fluorescent quantitative PCR primers is shown in Table 1.

### Western blot

After washing the cells 3 times with PBS, protein extraction kits (Solarbio Company, Beijing, China) were used to extract total cellular protein from preadipocytes. A 20 μg sample was taken for gel electrophoresis, then the remaining protein transferred to polyvinylidene fluoride membrane. Next, the membrane was incubated with antibodies against GAPDH (1:5,000, abcam, Cambridge, UK), CHRNG (1:500, Bioss, Beijing, China), PCNA (1:500, Santa Cruze, CA, USA), CCNB1 (1:1,000, Santa Cruze, USA), CCND2 (1:1,000, abcam, UK), PPARγ (1:1,000, abcam, UK), FABP4 (1:1,000, Santa Cruze, USA), FASN (1:10,000, abcam, UK) for 12 h at 4°C. Then the antibody was recovered, the membrane was rinsed with Tris buffered saline+Tween (TBST) solution 3 times, 10 min each time, the second antibody was diluted to the corresponding proportion with secondary antibody diluent, the incubation film was kept away from light on the shaker for 2 hours at room temperature, the membrane was rinsed with TBST solution 3 times, 10min each time, and chemiluminescence development.

### Statistical analysis

Data were analyzed using t-test in GraphPad Prism 6 software. The results were presented as mean±standard deviation, and p values of <0.05 were considered a statistically significant. We performed three replicates of each experiment.

## RESULTS

### Detection of *CHRNG* overexpression efficiency

**Figure 1:**
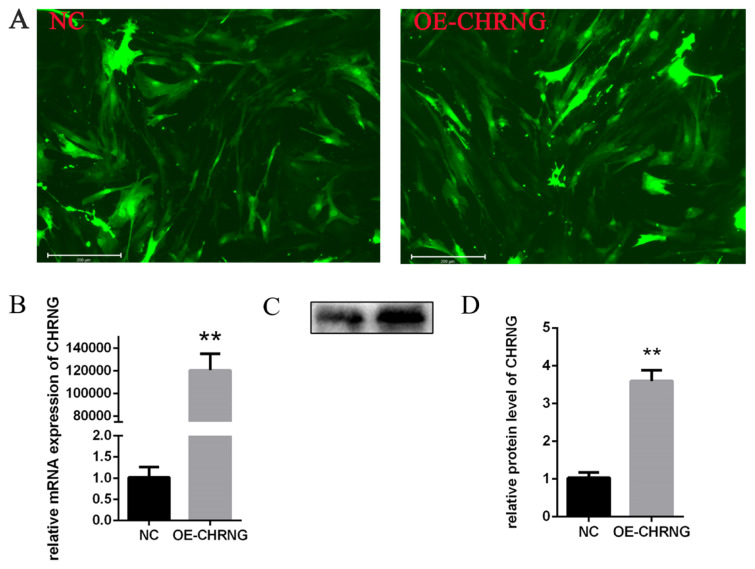
CHRNG overexpression efficiency test (A) 48 h infection efficiency of adipocytes transfected with adenovirus. Scale bar = 200 μm. (B) RT-qPCR detection of CHRNG overexpression efficiency. (C–D) CHRNG protein expression level of adenovirus-transfected preadipocytes and quantify. CHRNG, cholinergic receptor nicotinic gamma subunit; RT-qPCR, real-time fluorescence quantitative polymerase chain reaction. * p<0.05, ** p<0.01. Scale bar = 200 μm.

Objective shuttle plasmid *CHRNG* and adenovirus cytoskeleton were cotransfected into HEK293 cells to obtain recombinant virus *CHRNG* (Shanghai Heyuan Biotechnology Co. Ltd., Shanghai, China). At present, adipocytes were infected with adenovirus when they grew to about 80%. We observed the infection efficiency under a fluorescence microscope after 48 hours (Figure 1A), which was about 80%. At the same time, we detected the overexpression efficiency of *CHRNG*. Its mRNA level was up-regulated by about 120,000 times (Figure 1B), and we further detected and quantified the protein expression level (Figure 1C and 1D). We noticed that the expression levels of the two were inconsistent, because the transcription level and translation level was inconsistent. The results showed that the adenovirus could be used for follow-up research.

### Overexpression of *CHRNG* gene inhibits the proliferation of bovine preadipocytes

**Figure 2:**
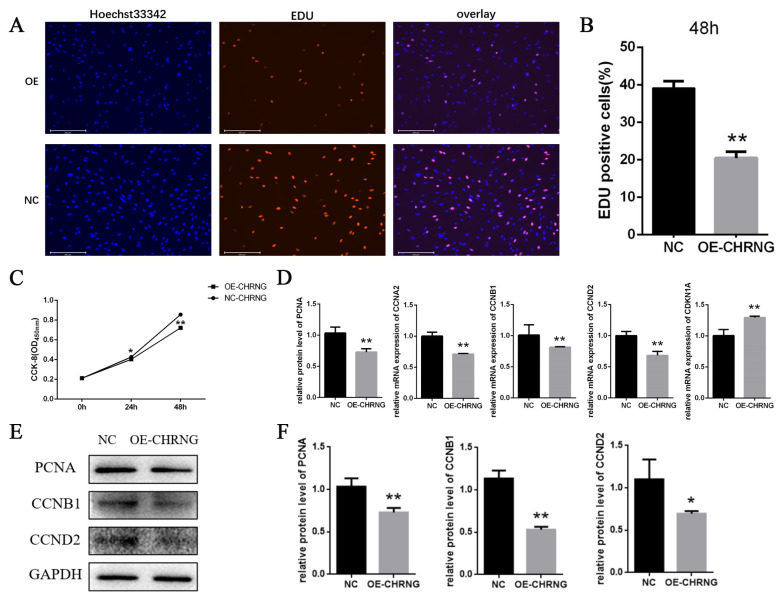
Overexpression of CHRNG inhibits the proliferation of bovine preadipocytes. (A–B) Following infection of preadipocytes with adenovirus, proliferating cells were labeled. Nuclei were stained with DAPI. Scale bar = 200 μm. Quantitative determination of the percentage of EdU+cells. (C) CCK-8 was used to detect cell proliferation. (D) Real-time quantitative polymerase chain reaction analysis of the mRNA expression levels of PCNA, CCNB1, CCNA2, CCND2, and CDKN1A after adenovirus-infected preadipocytes 48 h. (E) Western blot analysis on the expression of PCNA, CCNB1, and CCND2 after adenovirus-infected preadipocytes 48 h. (F) WB quantitative results. * p<0.05, ** p<0.01. CHRNG, cholinergic receptor nicotinic gamma subunit; DAPI, 4′,6-diamidino-2-phenylindole; CCK-8, cell counting Kit-8; PCNA, proliferating cell nuclear antigen; CCNB1, cyclin B1; CCNA2, cyclin A2; CCND2, cyclin D2; CDKN1A, cyclin dependent kinase inhibitor 1A. Scale bar = 200 μm.

To investigate the effect of overexpression of *CHRNG* gene on the proliferation, EdU analysis and cell counting Kit-8 (CCK-8) were performed to explore the effect of *CHRNG* on preadipocyte proliferation. As shown in Figures 2A and 2B, the mitotic activity of adipocytes in the treatment group was lower, and the number of EDU-positive cells was lower compared with the control group. The result of CCK-8 (Figure 2C) showed that the growth ability of the treatment group was significantly reduced, which was consistent with the results of EDU analysis. In order to confirm the effect of *CHRNG* on the proliferation of preadipocytes, the expression levels of some important cell regulatory factors were tested (Figure 2D). The mRNA expression of cyclin (proliferating cell nuclear antigen [*PCNA*]) and cyclin B1 (*CCNB1*), cyclin A2 (*CCNA2*), cyclin D2 (*CCND2*) was extremely significantly reduced, and the level of cyclin-dependent kinase inhibitor 1A (*CDKN1A*) was significantly increased. According to the Western Blot results, the protein expression levels of cyclin (PCNA), CCNB1, and CCND2 in the treatment group were lower than those in the control group (Figure 1E). The results were quantified (Figure 2F), the protein expression of PCNA and CCNB1 were extremely significantly reduced, and the protein expression of CCND2 was significantly reduced. These results collectively suggest that overexpression of *CHRNG* inhibits the proliferation of bovine preadipocytes.

### Overexpression of *CHRNG* gene inhibits the differentiation of bovine preadipocytes

**Figure 3:**
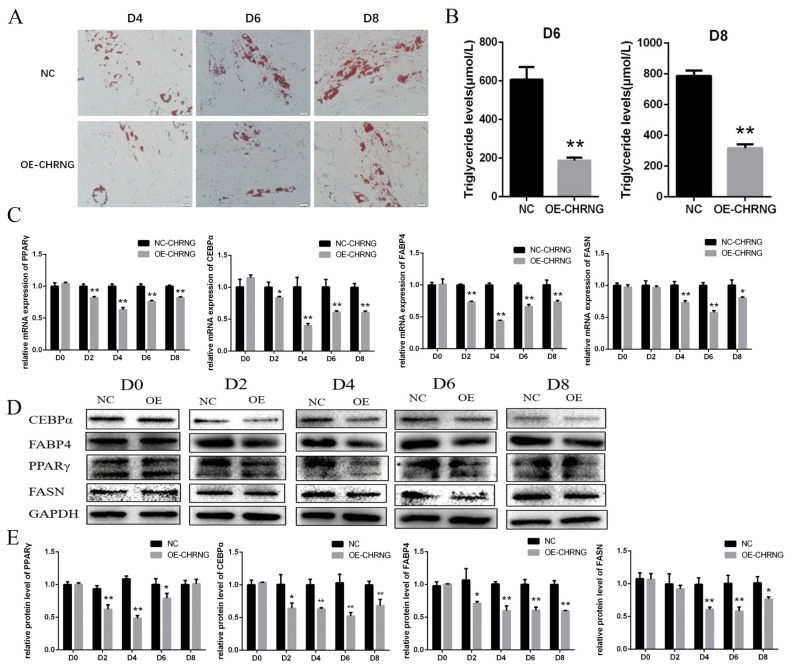
Overexpression of CHRNG inhibits the differentiation of bovine preadipocytes. (A) The preadipocytes were induced to differentiate after 48 h of oversxpression and oil red O staining was performed on differentiated D4, D6, and D8. Scale bar = 20 μm. (B) Depermination and quantification of triglyceride content of differentiated adipocytes (D6, D8) (C) Real-time quantitative polymerase chain reaction analysis of the mRNA expression levels PPARγ, C/EBPα, FABP4, FASN after induction of differentiation at D0, D2, D4, D6, D8. (D) Western blot analysis on the expression of PPARγ, C/EBPα, FABP4, and FASN. (E) WB quantitative results. * p<0.05, ** p<0.01. CHRNG, cholinergic receptor nicotinic gamma subunit; PPARγ, peroxisome proliferator activated receptor gamma; C/EBPα, CCAAT enhancer binding protein alpha; FABP4, fatty acid binding protein 4; FASN, fatty acid synthase.

To further investigate the effect of CHRNG on the differentiation of preadipocytes, oil red O, triglyceride determination, RT-qPCR and Western Blot were performed. Analysing the oil red O staining signals (Figure 3A), indicated that the total lipid accumulation of the overexpression group was significantly reduced, and the total amount of lipid accumulation was significantly less than that of the control group on different days of differentiation. In agreement with the result of oil red O, the triglyceride content of the treatment group was significantly lower on the sixth and eighth days of differentiation (Figure 3A). As shown in Figure 3C, the mRNA level of peroxisome proliferator activated receptor gamma (PPARγ) did not change significantly in D0 and D8, but decreased significantly in D2 and D4, and very significantly dropped in D6. The mRNA level of CCAAT enhancer binding protein alpha (C/EBPα) decreased significantly in D2, and very significantly decreased in D4, D6 and D8. The mRNA level of fatty acid binding protein 4 (FABP4) decreased significantly at D2, D4, D6, and D8, while the mRNA level of fatty acid synthase (FASN) decreased significantly at D4, D6, and D8. At the same time, we detected the protein expression of PPARγ, C/EBPα, FABP4, and FASN by Western Blot (Figure 3D) and quantified the results (Figure 3E). The protein expression of PPARγ in D2, D4, and D6 decreased significantly; the protein expression of C/EBPα and FABP4 decreased significantly in D2, and in D4, D6, and D8; the protein expression of FASN decreased significantly in D4 and D6, and significantly decreased in D8. The above results suggest that overexpression of CHRNG inhibits the differentiation of bovine preadipocytes.

## DISCUSSION

In livestock, the development of fat and muscle plays an important role in the formation of beef [17]. The process of preadipocyte proliferation and differentiation is the process of lipid accumulation [18]. This differentiation is the result of the synergistic effect of crucial transcription factors, especially PPARγ and C/EBPα [19]. At present, it has not been reported that *CHRNG* can regulate cell proliferation and differentiation, but this gene is a potential candidate gene screened by transcriptome sequencing in our laboratory under the co-culture system of preadipocytes and myoblasts.

Cell proliferation is regulated by cell cycle regulators [20]. Activation and aggregation of CDK and mitogen-dependent signaling pathways regulate adipocyte proliferation and maturation [21]. EDU proliferation experiment was performed to preliminarily judge the effect of CHRNG on bovine pre-adipocytes and we found that the number of EDU-positive cells in the treatment group was significantly less. We know that EdU can replace thymine (T) during DNA replication. Infiltrating into the DNA molecule being synthesized, it is preliminarily judged that the adipocyte proliferation ability of the treatment group was reduced, and the mitotic activity was lower. At the same time, CCK-8 test was carried out, and the conclusion was that the number of cells in the treatment group during the proliferation period was significantly lower than that of the control group, which is consistent with the result of EDU. To explore the possible mechanism of CHRNG affecting the proliferation of pre-adipocytes, RT-qPCR experiments and Western blot were performed. The results of RT-qPCR experiments showed that PCNA, CCNA2, CCNB1, and CCND2 were significantly reduced. As far as we know, the protein encoded by PCNA helps to increase the continuous ability of leader strand synthesis during DNA replication [22] and can interact with factors that regulate the cell cycle [23]. CCNB1 plays a key role in promoting the transition from G2 phase to mitosis [24]. While CCNA2 controls the G1/S and G2/M transition phases of the cell cycle [25]. Due to the decrease in the expression of these genes, the cycle transformation of the precursor adipocytes is inhibited, resulting in the inhibition of the cell cycle, and further inhibiting the proliferation of the precursor adipocytes. Western blot results indicate that the protein expression of PCNA, CCNB1, and CCND2 was also extremely significantly reduced, which is consistent with the results of RT-qPCR analysis. These marker genes affected by CHRNG overexpression are all related to cell cycle signaling pathways, so the inhibition of preadipocyte proliferation is mainly caused by changes in its cell cycle progression. As described above, overexpression of the *CHRNG* gene in preadipocytes inhibits cell proliferation.

Adipocytes play a core role in maintaining the dynamic balance of metabolism in the body [26], adipogenesis is strictly regulated and involves a complex network of transcription factors that play a role at different time points of differentiation [27,28]. By staining with oil red O, we found that the accumulation of lipid droplets slowed down after CHRNG was overexpressed in preadipocytes and the size of lipid droplets was lower. We preliminarily judged that preadipocytes differentiation was inhibited by CHRNG. At the same time, the content of triglycerides was tested on the sixth and eighth days of differentiation, and the conclusions obtained were consistent with oil red O. To explore the possible mechanism of CHRNG affecting the differentiation of preadipocytes, RT-qPCR experiments and Western blot were performed. RT-qPCR experiment results showed that the expression of *PPAR-γ*, *C/EBPα*, *FABP4*, and *FASN* is extremely significantly reduced. We know that the differentiation of preadipocytes requires PPARγ and C/EBPs [29], during the differentiation process, PPAR-γ and CEBP-α will activate some target genes, such as *FAS*, *FABP4*, stearoyl-CoA desaturase (*SCD*), etc, and coordinately activate the final stage of differentiation, thereby creating and maintaining the adipocyte phenotype [30]. This result confirms that *CHRNG* can inhibit the differentiation of preadipocytes by regulating transcription factors such as PPAR-γ and CEBP-α. But it is the protein that really plays a role in the cell, so Western Blot experiments were performed to detect and quantify the protein expression levels of PPAR-γ, C/EBPα, FABP4, and FASN, and found that the expression levels of these proteins have varying degrees. Of particular note is PPAR-γ, which is the most important factor for the differentiation of preadipocytes, and this gene is involved in the MAPK signaling pathway. CHRNG may further inhibit the differentiation of preadipocytes through this pathway. The decrease in cytotoxicity further proves that CHRNG influences the key factors regulating fat differentiation, but its specific regulation mechanism needs to be further studied.

Adipose tissue development process is extremely compli cated, which is completed by the joint action of many genes [8]. The effects of genes on the proliferation and differentiation of preadipocytes are opposite, that is, inhibiting proliferation and promoting differentiation or promoting proliferation and inhibiting differentiation. However, our research results show that overexpression of the *CHRNG* gene inhibits the proliferation and differentiation of preadipocytes. Through experiments, we found that CHRNG has an inhibitory effect on the proliferation and differentiation of adipocytes. If subsequent experiments can knock out this gene through CRISPR-Cas9 and other technologies to prove that it can promote cell proliferation and differentiation, and can affect bovine intramuscular fat deposition, then it can be applied in gene editing breeding or transgenic breeding. At the same time, it is also possible to search for the single nucleotide polymorphism loci related to fat deposition on the gene and use this gene to carry out marker-assisted selection breeding. In summary, through research we have found that overexpression of CHRNG inhibits the proliferation and differentiation of preadipocytes, which has laid a certain theoretical foundation for molecular breeding. At the same time, the research results show that it has an important regulatory effect on the growth and development of Qinchuan cattle muscle tissue. Through in-depth functional research, it can be used in the practice of molecular breeding of beef cattle.

## Figures and Tables

**Table 1:** Primer specific information

Gene name	GenBank No.	Primer name	Primer sequence (5′→3′)	Product length
*GAPDH*	NM_001034034.2	*GAPDH*-F	AGTTCAACGGCACAGTCAAGG	124
		*GAPDH*-R	ACCACATACTCAGCACCAGCA	
*CHRNG*	NM_174273.2	*CHRNG*-F	CAGTCCCAGACCTACAGCAC	115
		*CHRNG*-R	CCCACTCCCCATTTTCTGTGA	
*PCNA*	NM_001034494.1	*PCNA*-F	CCTTGGTGCAGCTAACCCTT	94
		*PCNA*-R	TTGGACATGCTGGTGAGGTT	
*CCNB1*	NM_001045872.1	*CCNB1*-F	TACCCATTCACCATTATCAA	103
		*CCNB1*-R	ACTAACTATGCTGGACTACGA	
*CCNA2*	NM_001075123.1	*CCNA2*-F	GCAGCCTTTCATTTAGCACTCT	155
		*CCNA2*-R	ATTGACTGTTGTGCGTGCTG	
*CCND2*	NM_001076372.1	*CCND2*-F	CGCCAGGTTCCATTTCA	77
		*CCND2*-R	CCGACAACTCCATCAAGC	
*CDKN1A*	XM_005223326.4	*CDKN1A*-F	GACCAGCATGACAGATTTCTACCA	144
		*CDKN1A*-R	TGAAGGCCCAAGGCAAAAG	
*PPARγ*	NM_181024.2	*PPARγ*-F	GACGACAGACAAATCACCGT	155
		*PPARγ*-R	CTTCCACGGAGCGAAACTGA	
*C/EBPα*	NM_176784.2	*C/EBPα*-F	ATCTGCGAACACGAGACG	73
		*C/EBPα*-R	CCAGGAACTCGTCGTTGAA	
*FABP4*	NM_174314.2	*FABP4*-F	TGAGATTTCCTTCAAATTGGG	101
		*FABP4*-R	CTTGTACCAGAGCACCTTCATC	
*FASN*	NM_001012669.1	*FASN*-F	GGCAAACGGAAAAACGGTGA	183
		*FASN*-R	CTTGGTATTCCGGGTCCGAG	

*GAPDH*, glyceraldehyde-3-phosphate dehydrogenase; *CHRNG*, cholinergic receptor nicotinic gamma subunit; *CCK-8*, cell counting Kit-8; *PCNA*, proliferating cell nuclear antigen; *CCNB1*, cyclin B1; *CCNA2*, cyclin A2; *CCND2*, cyclin D2; *CDKN1A*, cyclin dependent kinase inhibitor 1A; *PPARγ*, peroxisome proliferator activated receptor gamma; *C/EBPα*, CCAAT enhancer binding protein alpha; *FABP4*, fatty acid binding protein 4; *FASN*, fatty acid synthase; *SCD*, stearoyl-CoA desaturase.
